# Communication Difficulties Among Older Adults With Different Degrees of Cognitive Impairment

**DOI:** 10.1002/brb3.71372

**Published:** 2026-04-06

**Authors:** Hui‐Chuan Hsu, Chyi‐Huey Bai, Shu‐Nu Chang‐Lee, Jiun‐Yi Wang, Sheng‐Feng Lin, Yi‐Wen Liu

**Affiliations:** ^1^ School of Public Health Taipei Medical University New Taipei Taiwan; ^2^ Department of Public Health School of Medicine Taipei Medical University Taipei Taiwan; ^3^ Department of Long‐Term Care National Quemoy University Kingmen Taiwan; ^4^ Department of Healthcare Administration Asia University Taichung Taiwan; ^5^ School of Medicine Taipei Medical University Taipei Taiwan; ^6^ Department of Electrical Engineering National Tsing Hua University Hsinchu Taiwan

**Keywords:** cognitive impairment, communication, dementia, quality of life

## Abstract

**Background:**

The prevalence of dementia has been increasing, and communication difficulties among people with cognitive impairment are burdensome and frustrating for both older adults and caregivers. However, subjective communication difficulties have rarely been examined in the context of communication as a two‐way interaction. The purpose of this study was to examine communication difficulties among older adults with various degrees of cognitive impairment and to explore related factors and their associations with quality of life.

**Methods:**

In this cross‐sectional survey study, face‐to‐face interviews were conducted to collect data from older adults aged 50 years or older with various degrees of cognitive impairment in the community (hospitals or daycare centers) or in long‐term care institutions. In total, 133 people were included in the analysis.

**Results:**

Five dimensions of communication difficulties were extracted via a factor analysis: communication stress, wording difficulties, negative interactions, family/friend chatting, and family/friend adaptations. Family/friend adaptation difficulties were related to a lower likelihood of having a good quality of life (odds ratio = 0.562, *p* < 0.01). Greater total communication difficulties were related to a diagnosis of dementia (B = 2.991; *p* < 0.05), subjective memory complaints (B = 2.562; *p* < 0.05), and lower social support (B = −1.064; *p* < 0.001). Among the dimensions of communication difficulties, communication stress and wording difficulties were related to cognitive function‐related variables, whereas negative interactions, family/friend chatting, and family adaptations were related to social support.

**Conclusions:**

Older adults may care more about negative interactions and how family/friends interact with them than about cognitive difficulties, and social support is essential for reducing communication difficulties. Caregivers should learn how to interact with people with cognitive impairment to reduce difficulties and the burden of caregiving.

## Graphical abstract

This study examined communication difficulties among older adults with various degrees of cognitive impairment and to explore the related factors and their associations with quality of life. Total communication difficulties and family/friend adaptation difficulties were related to poor quality of life. Communication difficulties were related to a diagnosis of dementia, subjective memory complaints, and lower social support.

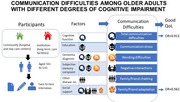

## 1 Introduction

There are 55.2 million people with dementia worldwide, and the global cost of medical and family care related to dementia is expected to be more than $2.8 trillion by 2030 (WHO 2022). One of the symptoms of impaired cognition is communication difficulties, which may appear in the early stage of cognitive impairment before a dementia diagnosis. Communication difficulties not only reduce the quality of life and affect the utilization of health care for people with cognitive impairment but also prove to be burdensome for family caregivers and formal caregivers. In recent years, studies have explored relationships of language and voice performance with dementia (Filiou et al. 2020; Mueller et al. 2018; Wang et al. 2021). However, past studies rarely explored the problems associated with communication difficulties among people with cognitive impairment, especially their frustration with communication. Nevertheless, communication difficulties are not just a health condition for people with cognitive impairment. Caregivers may lack awareness or proper skills and may therefore increase communication difficulties (Lanzi et al. 2022). In this study, we aimed to assess communication difficulties among people with different degrees of cognitive impairment and explore factors related to communication difficulties and the effects of those difficulties on quality of life.

Communication difficulties are often observed in people with cognitive impairment (Machiels et al. 2017). Symptoms include delayed recall of names and places, frequent and long pauses during a conversation, the use of vague words or pronouns in sentences, the inability to name things correctly, the use of improper words, the repetition of content or questions, and so forth (Savundranayagam et al. 2005). People with cognitive impairment are still emotionally sensitive. According to a previous study (Alsawy et al. 2019), although people with cognitive impairment have difficulties with language, their families strive to connect with them, and they may still feel meaningful moments and understand their family's feelings. However, when families try to consciously compensate for communication difficulties or use elderspeak (Zhang et al. 2020), people with cognitive impairment may feel stressed or frustrated because they do not want to reveal their inability to engage in conversation. People with cognitive impairment may try to avoid or pretend that there is no problem with communication during dialogue.

Existing studies on communication difficulties have primarily assessed caregiving burdens from the perspective of caregivers (Alsawy et al. 2019; Olthof‐Nefkens et al. 2021; Petrovsky et al. 2020; Savundranayagam et al. 2005; Zhang et al. 2020). Few scales have assessed communication difficulties among people with cognitive impairment and focused only on language abilities (Bryan et al. 2001). However, communication is a two‐way interaction. Communication difficulties are more than just the responsibility of people with cognitive impairment, and family or caregivers should take on more responsibility when communicating (Braithwaite Stuart et al. 2022). The use of negative or improper communication methods among family members and formal caregivers (Gitlin et al. 2010; Petrovsky et al. 2020) may increase the difficulty and burden of caregiving, as well as the frustration of people with cognitive impairment (Savundranayagam et al. 2005; Gitlin et al. 2010). However, most studies examined communication difficulties qualitatively, and existing assessments focused on language abilities but less considered the conversation partners (Dooley and Walshe 2019).

In Taiwan, 17.99% of older people had mild cognitive impairment (MCI), and 7.54% had dementia in 2022 (Taiwan Alzheimer's Disease Association 2023). Care for people with cognitive impairment is urgently needed in Taiwan and all aging societies. The purpose of this study was to identify communication difficulties among middle‐aged and older adults with various levels of cognitive function in Taiwan and examine associations of communication difficulties with related factors and their effects on quality of life. The findings are expected to provide implications for developing useful strategies for caring for people with cognitive impairment and improving their psychological well‐being.

## 2 Methods

### 2.1 Data and Sample

Data were collected via face‐to‐face questionnaires administered from October 2024 to May 2025. We used purposive sampling from the community (hospitals or daycare centers) and long‐term care (LTC) institutions to obtain participants with various degrees of cognitive impairment. Participants were recruited from four residential LTC facilities and two community‐based daycare centers in Taichung and the neurology department of one hospital in New Taipei City. The inclusion criteria were being aged ≥ 50 years, being able to communicate with others, being able to make decisions, and being willing to participate in the study. Exclusion criteria included having severe dementia or an inability to communicate (due to loss of consciousness, severe cognitive impairment, or the inability to talk) or not having the autonomy to show willingness to participate in the study. The study project was approved by institutional review boards before it was conducted. All participants signed an informed consent form before being allowed to participate in the study. The study was conducted in accordance with guidelines of the Declaration of Helsinki. G*Power 3.1.9.2 software was used to calculate the necessary sample size. The test family was the *F*‐test; statistical test was a linear multiple‐regression fixed model *R* ^2^ deviation from zero: point biserial model (the type of power was a priori). We set the effect size to 0.3, *α* to 0.05, power to 0.95, and number of predictors to 11. Then the minimum sample size was 95. The pilot test sample had to be 5%, and thus we invited seven persons to conduct the pilot test. In total, 136 people participated in the formal study, and 133 people completed the questionnaires.

Data were collected through a questionnaire interview and voice recording in the picture description section. A questionnaire was drafted on the basis of a literature review. Next, experts with backgrounds in gerontology, LTC, epidemiology, and neurology were invited to review the questionnaire and provide suggestions for modification to ensure the content validity and face validity. The questionnaire was pretested, and sentences were accordingly modified. The final questionnaire was subsequently confirmed for formal use in the study. The interviewers were well trained before the interviews were conducted.

### 2.2 Assessment of Communication Difficulties

Communication difficulties were measured by 18 items in a design based on the previous literature (Alsawy et al. 2019; Gitlin et al. 2010; Petrovsky et al. 2020; Savundranayagam et al. 2005). At the conceptual level, items were related to wording difficulty, conversation stress with others, interactions with family/formal caregivers/community people, and family members’ reactions and adaptations to cognitive impairment in older adults. Each item was scored from 0 to 3, respectively indicating “never,” “occasionally,” “sometimes,” or “all the time.” Scores of positive descriptions were coded in reverse, so a higher score indicated greater difficulties (please see Table S1). The internal consistency Cronbach's alpha value of the scale of communication difficulties for all the items was 0.804, indicating high internal consistency. The total degree of communication difficulties was assessed by the summed score of all items.

Next, five dimensions were extracted through an exploratory factor analysis to explore the constructs: communication stress, wording difficulties, negative interactions (with professionals or the community), family/friend chatting difficulties, and family/friend adaptation difficulties (please see Table S2). The explained variance was 67.7%. The internal consistency Cronbach's alpha values of the five factors were 0.833 (communication stress), 0.793 (wording difficulties), 0.735 (negative interactions), 0.867 (family/friends chatting), and 0.890 (family/friend adaptations), indicating high internal consistency within each dimension. Next, the original scores of the items within the dimension were summed and divided by the number of items to obtain the average scores of the dimension, and difficulties across dimensions were compared.

### 2.3 Assessment of Cognitive Function

Cognitive function was measured by the traditional Chinese version of the Montreal Cognitive Assessment (MoCA) (Nasreddine et al. 2005), a widely used tool for assessing cognitive function. The total score and scores of the dimensions of cognitive function in the MoCA were used in this study (Sala et al. 2020): short‐term memory, calculation (subtraction), time and place orientation, attention (digital span forward, digital span backward, attention, and sentence repetition 1 and 2), visuospatial executive function (trail making, cube, and clock hand and shape), language (naming camel, lion, and rhino, and verbal fluency), and abstraction (abstraction 1 and 2).

### 2.4 Measures of Other Variables

Quality of life was used to examine the effects of communication difficulties. Quality of life was defined by asking the participant to rate his or her quality of life as poor, fair, good, or excellent. The item was coded as poor/fair (0) or good/excellent (1). Covariates used in this study included age, sex (male/female), education (ordinal from 0 to 5, indicating from illiterate to graduate school and above), source of each case (hospital/LTC facility), dementia diagnosis (no/yes), number of chronic diseases (total number of morbidities of the following diseases: hypertension, diabetes, hyperlipidemia, heart diseases, stroke, ophthalmological diseases, hearing problems, ulcers, gout disease, arthritis, respiratory diseases, kidney diseases, dialysis, liver diseases, cancers, psychological diseases, broken bones, and reproductive diseases), subjective memory complaint (by asking “Do you think you have memory problems?” with answers of “no problem” or “occasionally forgetful” (recoded as “no”) or memory is often poor or always poor (recoded as “yes”), hearing (clear/unclear), and social support. Social support was measured using scores for three items about satisfaction with care provided by the family, satisfaction with care provided by the family when sick, and satisfaction with care provided by friends/neighbors or roommates. Each item was scored from 0 to 5, and the total score ranged 0–15; a higher score indicated better social support.

### 2.5 Analysis

Descriptive analysis, bivariate analysis, factor analysis, linear regression, and logistic regression were conducted. Pearson's correlations were used to examine correlations of communication difficulties with covariates to select significant variables for a multivariate analysis, and one‐way analysis of variance (ANOVA) was used to determine differences in communication difficulties according to the level of cognitive impairment. Factor analysis was used to explore the dimensions of communication difficulties, and the total score of the scale and scores by dimension were used to analyze related factors. The linear regression was used to examine factors related to communication difficulties, and the logistic regression was used to examine the effects of communication difficulties on quality of life. The collinearity of the covariates was tested. The analysis was conducted using SPSS 22.00 (IBM, Armonk, New York, USA).

## 3 Results

Table 1 gives a description of the sample. Among the participants, 54.1% were female, and 45.9% were male; 42.9% were from LTC institutions, and 57.1% were from the community (hospitals or daycare centers). Approximately two‐thirds of the older participants had a low level of education (primary high school or lower). Among the participants, 19.7% had a diagnosis of dementia.

**TABLE 1:** Description of the participants.

Variables	Mean (SD) or %
Age	71.99 (7.67)
50–64	12.8%
65–74	54.9%
75+	32.3%
Sex	
Female	54.1%
Male	45.9%
Source of cases	
Institutions	42.9%
Community	57.1%
Educational level (ordinal 0–4)	1.88 (1.18)
Illiterate	6.0%
Elementary school or informal education	45.1%
Primary high school	16.5%
Senior high school	19.5%
College/university and above	12.8%
Diagnosis of dementia	
No	80.3%
Yes	19.7%
Other chronic disease numbers (0–19, excluding dementia)	2.61 (1.18)
Subjective memory complaint	
No	59.4%
Yes	40.6%
Hearing	
Clear	87.1%
Unclear	12.9%
Social support (0–15)	11.34 (2.45)
Quality of life	
Poor/fair	66.9%
Good/excellent	33.1%
Cognitive function (MoCA score) (0–30)	15.15 (7.35)
Normal (MoCA score 26–30)	8.3%
Mild impairment (MoCA score 18–25)	34.6%
Moderate impairment (MoCA score 10–17)	32.3%
Severe impairment (MoCA score < 10)	24.8%
Cognitive function (MoCA) by dimension	15.15 (7.35)
Visual spatial execution (0–5)	2.38 (1.80)
Attention (0–5)	2.89 (1.40)
Calculation (0–3)	1.94 (1.16)
Language (0–4)	2.43 (1.38)
Abstract concept (0–2)	0.74 (0.78)
Delayed recall memory (0–5)	0.78 (1.41)
Time/space orientation (0–6)	3.99 (1.97)
Total communication difficulties	11.42 (6.72)
Conversation stress (0–3)	0.22 (0.44)
Wording difficulties (0–3)	0.97 (0.75)
Negative interaction (0–3)	0.23 (0.41)
Family/friends chatting difficulties (0–3)	1.05 (1.00)
Family/friend adaptation difficulties (0–3)	1.61 (1.21)

*Note*: = 133. Missing cases were excluded listwise.

**FIGURE 1:**
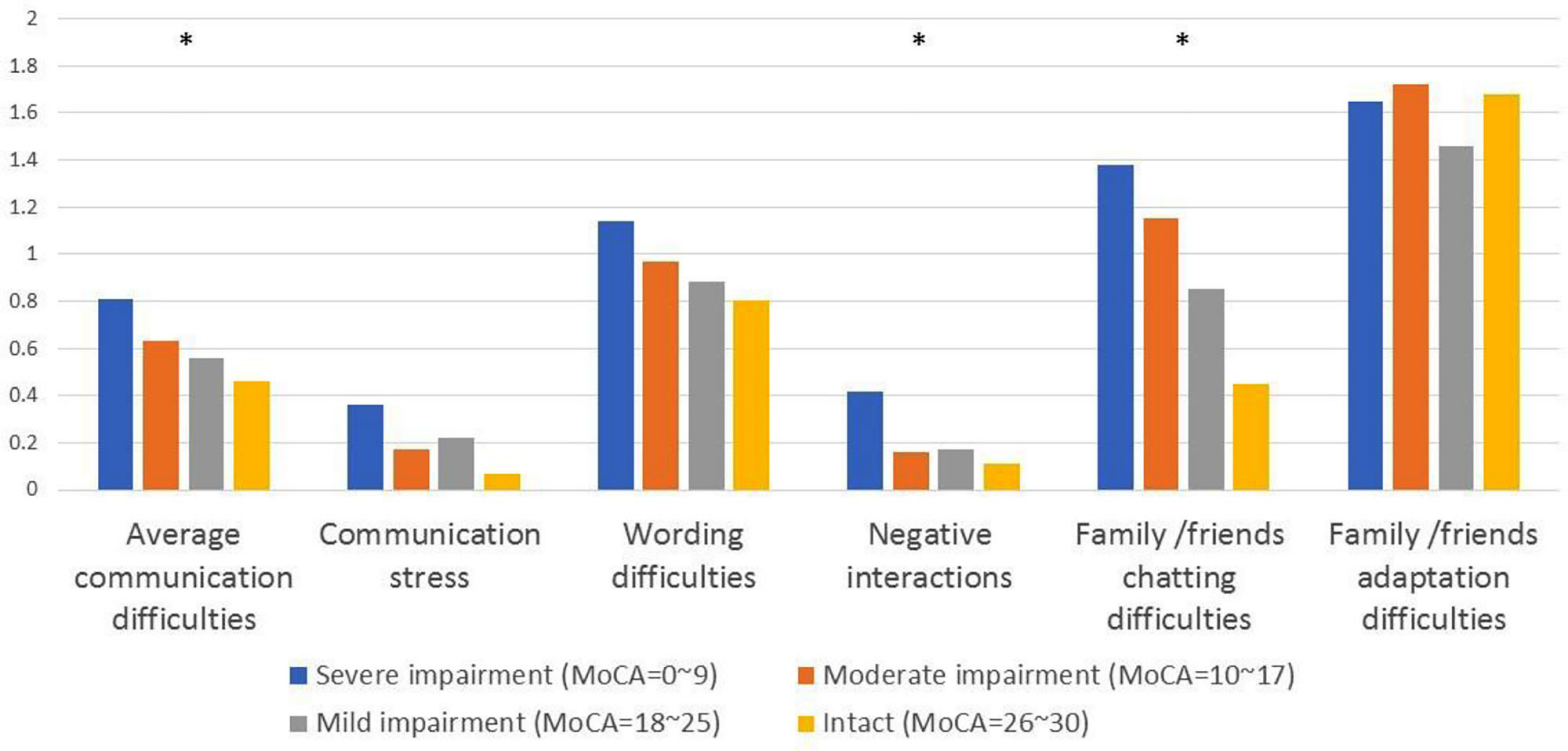
Communication difficulties and dimensions by cognitive impairment degree.

The total number of communication difficulties and their dimensions across different levels of cognitive impairment are shown in Figure 1. The total communication difficulty score was averaged across the items. Results show that the greater the level of cognitive function, the lower the degree of communication difficulties. There were significant differences in total communication difficulties, negative interactions, and family/friend chatting difficulties.

Table S3 shows results of the bivariate analysis of communication difficulties with respect to cognitive function and covariates by Pearson's correlations. Total communication difficulties were related to overall cognitive function (*r* = −0.269, *p* < 0.01) and multiple dimensions, including attention, calculation, language, abstraction, and time–space orientation. Only one dimension of cognitive function was related to communication stress (time–space orientation, *r* = −0.211) and wording difficulties (attention, *r* = −0.170). None of the dimensions of cognitive function were related to family/friend adaptations. Negative interactions and family/friend chatting difficulties were related to multiple dimensions of cognitive function, but none of the dimensions was related to family/friend adaptation difficulties. A dementia diagnosis and subjective memory complaints were related to increased communication stress and wording difficulties. Hearing was related to more difficulties with family/friend chatting. Greater social support was related to fewer total communication difficulties and less difficulty in negative interactions, family/friend chatting, and family/friend adaptations.

Associations between communication difficulties and quality of life are shown in Table 2. M1 shows the effect of total communication difficulties on quality of life. Individuals with fewer communication difficulties (odds ratio [OR] = 0.913, *p* < 0.05), fewer other diseases (OR = 0.673, *p* < 0.05), and greater social support (OR = 1.817, *p* < 0.001) were more likely to have a good quality of life. In M2, communication difficulties were assessed by dimension. Family/friend adaptation difficulties were related to a lower likelihood of having a good quality of life (OR = 0.562, *p* < 0.01), while other diseases and social support were still significant. Table 3 shows associations of communication difficulties (and by dimension) with related factors according to the linear regression. Greater total communication difficulties were related to a diagnosis of dementia (B = 2.991; *p* < 0.05), subjective memory complaints (B = 2.562; *p* < 0.05), and lower social support (B = −1.064; *p* < 0.001). High levels of communication stress were related to high levels of education (B = 0.080; *p* < 0.05), a diagnosis of dementia (B = 0.340; *p* < 0.01), and subjective memory complaints (B = 0.227; *p* < 0.01). Greater wording difficulty was related to having dementia (B = 0.444; *p* < 0.05) and subjective memory complaints (B = 0.461; *p* < 0.01). More‐negative interactions were related only to lower social support (B = −0.055, *p* < 0.001). Greater difficulties in family/friend chatting were related to lower levels of cognitive function (B = −0.032, *p* < 0.05), more other diseases (B = −0.093, *p* < 0.05), and lower levels of social support (B = −0.175, *p* < 0.001). Greater difficulties in family/friend adaptation were only related to lower social support (B = −0.110, *p* < 0.005).

**TABLE 2:** Associations of communication difficulties with quality of life.

Variables	M1. Good quality of life odds ratio (95% CI)	M2. Good quality of life odds ratio (95% CI)
Communication difficulties (total)	0.913 (0.836–0.997)*	—
Communication difficulties by dimension		
Communication stress	—	0.506 (0.098–2.601)
Wording difficulties	—	1.262 (0.603–2.642)
Negative interactions (with professionals or the community)	—	0.403 (0.046–3.526)
Family/friend chatting difficulties	—	0.773 (0.407–1.469)
Family/friend adaptation difficulties	—	0.562 (0.365–0.865)**
Age	0.993 (0.922–1.069)	0.990 (0.915–1.071)
Sex (male)	2.126 (0.788–5.738)	2.805 (0.948–8.300)
Education	0.783 (0.480–1.277)	0.680 (0.396–1.167)
Case source (community)	1.518 (0.481–4.794)	1.410 (0.379–5.249)
Dementia (yes)	2.386 (0.640–8900)	1.649 (0.383–7.091)
Other disease number	0.673 (0.480–0.943)*	0.591 (0.400–0.883)*
Hearing (unclear)	0.624 (0.126–3.901)	0.441 (0.074–2.644)
Social support	1.817 (1.323–2.497)***	1.863 (1.299–2.673)**
Model fit	−2 log likelihood = 114.918, chi‐square = 49.442 (df = 9)***	−2 log likelihood = 107.722, chi‐square = 56.588 (df = 13)***

*Note*: Constant is omitted from the table. Reference groups: quality of life (poor), sex (female), case source (institution), dementia diagnosis (no), hearing (clear); other variables were ordinal or continuous. Analysis by logistic regression.

*p < 0.05, **p < 0.01, ***p < 0.001.

**TABLE 3:** Communication difficulties and its dimensions with related factors by linear regression.

Variables	Total communication difficulties	Communication stress	Wording difficulties	Negative interactions	Family/friends chatting difficulties	Family/friend adaptation difficulties
Constant	23.567 (6.712)**	0.920 (0.462)*	0.283 (0.803)	0.822 (0.442)	3.518 (0.942)***	3.344 (1.323)*
Cognitive function	−0.091 (0.110)	−0.005 (0.008)	0.018 (0.013)	−0.008 (0.007)	−0.032 (0.015)*	−0.016 (0.022)
Age	−0.016 (0.080)	−0.007 (0.005)	0.005 (0.010)	0.002 (0.005)	−0.002 (0.011)	−0.002 (0.016)
Sex (male)	0.436 (1.123)	−0.050 (0.077)	0.001 (0.134)	−0.041 (0.074)	0.067 (0.158)	0.378 (0.221)
Education	0.215 (0.561)	0.080 (0.039)*	−0.014 (0.067)	0.043 (0.037)	0.043 (0.079)	−0.214 (0.111)
Case source	−1.410 (1.458)	−0.086 (0.100)	−0.135 (0.174)	−0.131 (0.096)	0.275 (0.205)	0.383 (0.287)
Dementia (yes)	2.991 (1.459)*	0.340 (0.100)**	0.444 (0.175)*	0.068 (0.096)	−0.190 (0.205)	−0.223 (0.288)
Subjective memory complaint	2.562 (1.138)*	0.227 (0.078)**	0.461 (0.136)**	−0.012 (0.075)	−0.117 (0.160)	−0.059 (0.224)
Other disease number	0.397 (0.309)	−0.026 (0.021)	0.072 (0.037)	0.012 (0.020)	0.093 (0.043)*	−0.01 (0.061)
Hearing (unclear)	0.064 (1.679)	−0.105 (0.116)	0.073 (0.201)	−0.039 (0.111)	0.343 (0.236)	−0.098 (0.331)
Social support	−1.064 (0.222)***	−0.022 (0.015)	−0.027 (0.027)	−0.055 (0.015)***	−0.175 (0.031)***	−0.110 (0.044)*
*R* square	0.287	0.231	0.172	0.189	0.359	0.149

*Note*: Analysis by linear regression. Reference groups: quality of life (poor/fair), sex (female), case source (institution), dementia diagnosis (no), subjective memory complaint (no), hearing (clear); other variables were ordinal or continuous.

*p < 0.05, **p < 0.01, ***p < 0.001.

## 4 Discussion

In this study, we explored communication difficulties among older adults with various degrees of cognitive function, related factors, and effects of communication difficulties on quality of life. Five dimensions of communication difficulties were extracted: communication stress, wording difficulties, negative interactions, family/friend interactions, and family/friend adaptations. A multivariate analysis revealed that greater communication difficulties were related to a diagnosis of dementia, subjective memory complaints, and lower levels of social support. Among the dimensions of communication difficulties, communication stress and wording difficulties were more related to cognitive function‐related variables, whereas negative interactions, family/friend chatting, and family adaptations were more related to social support. Having fewer communication difficulties was related to a greater likelihood of having a good quality of life.

Few studies have assessed subjective communication difficulties among people with cognitive impairment, and related studies primarily focused on language abilities among people with dementia (Bryan et al. 2001; Dooley and Walshe 2019). In terms of subjective difficulties, frustration and communication difficulties were emphasized by participants with cognitive impairment but not from the expert's viewpoint. Self‐reported communication difficulties represent difficulties that are most important to people with cognitive impairment. In particular, we emphasized that communication is a two‐way interaction and that counterparts of people with cognitive impairment are also involved in communication. The extracted dimensions of communication difficulties were not only about wording difficulties and communication stress but also related to the counterparts in the communication, that is, negative interactions, family/friend chatting, and family/friend adaptations. These results serve as a response to part of the research goals and highlight the importance of developing communication strategies (Braithwaite Stuart et al. 2022). Our study provides a tool for systematically assessing subjective communication difficulties among people with cognitive impairment, which may be useful for exploring communication difficulties among people with different stages of cognitive impairment.

Compared to previous studies (Bryan et al. 2001; Dooley and Walshe 2019), the constructs of communication difficulties in our study showed the importance of family interactions, and two constructs about family interactions were extracted. Family‐centered culture is emphasized in the culture of Taiwan, especially for the older cohorts. Although older adults live in LTC institutions, they still need love and care from their family based on qualitative data in this study. Thus, the scale of communication difficulties may be displayed and can be applied to the strong needs of family support by older adults with cognitive impairment, especially in a family‐centered culture such as Taiwan. Meanwhile, the languages of the participants were Mandarin and/or Taiwanese. Wording difficulties associated with various degrees of cognitive impairment may also differ with other languages in other cultures.

In the bivariate analysis, overall cognitive function was negatively related to total communication difficulties as well as the dimensions of negative interactions and family/friends chatting. When controlling for covariates, cognitive function was still negatively related to family/friend chatting. On the one hand, when older adults have more‐severe cognitive impairment, they have more difficulties communicating with caregivers and people in the community to express their needs. On the other hand, family and formal caregivers and even people in the community might not know how to interact or might refuse to interact with people with cognitive impairment, or they may stigmatize people with cognitive impairment; thus, the communication approach may be discriminative. All these factors may increase negative interactions and reduce chatting with people with cognitive impairment; thus, communication difficulties increase.

However, difficulties associated with communication stress and wording were not strongly related to cognitive dimensions. It is also possible that older adults with cognitive impairment are not aware that their language ability is declining unless they perceive negative interactions and family chatting difficulties, or perhaps interactions with caregivers and neighbors in the community are more important to older adults than their self‐rated cognitive problems in daily life. Our results also revealed that greater difficulties with family/friend adaptations were significantly related to a lower quality of life. This finding indicates that the symptoms of cognitive decline do not worsen quality of life but rather that the environment and whether family and friends are willing to provide companionship and strive to adapt to their cognitive decline are factors that contribute to a decline in quality of life.

With respect to factors related to the five dimensions of communication difficulties, communication stress and wording difficulties were more related to the level of cognitive function, including educational level, a dementia diagnosis, and subjective memory complaints. However, in dimensions involving the counterparts of older adults (i.e., negative interactions, family/friend chatting, and family/friend adaptations), social support was more significant. On the one hand, when caregivers have greater difficulties chatting with people with dementia or lack communication adaptation skills, they may be reluctant to provide social support and even show discriminatory attitudes toward people with dementia; thus, older adults are more likely to perceive negative interactions. When formal or informal caregivers are more familiar with older adults with cognitive impairment and are willing to communicate with them (Alsawy et al. 2019), they adapt to the cognitive decline of older adults; thus, the communication difficulties of the older adults decrease. On the other hand, older adults with greater communication difficulties may be unable to perceive and express social support very well. Thus, they might not only misunderstand social support from caregivers but also frustrate their caregivers during the provision of social support. In fact, harsh communication is related to family caregiving burdens and depression (Petrovsky et al. 2020). Thus, family or formal caregivers should learn how to communicate with and adapt to people with cognitive impairment, which is beneficial not only for the older adults with cognitive impairment but also for family caregivers. For formal caregivers or caring staff in LTC institutions, good communication begins with making connections from daily contact with residents to increase trust and partnership (Hoek et al. 2021). Person‐centered communication training for formal caregivers is necessary (Savundranayagam et al. 2020); this training includes first observing, analyzing, and diagnosing communication problems between people with dementia and formal caregivers, and then providing suggestions for caregivers to improve their communication skills.

### 4.1 Limitations

There are several strengths of this study. First, the assessment of subjective communication difficulties considered not only language abilities but also interactions between counterparts who communicate with people with cognitive impairment. The caregiver's role and communication strategies were particularly highlighted in this communication. Second, we included participants with different degrees of cognitive impairment rather than only comparing healthy participants and patients with dementia. Gradual changes in communication difficulties could thus be differentiated.

There are several limitations of this study. First, the sample size was relatively small, which limited the covariates we could use in the multivariate analysis. Social support was also measured as a single dimension without being separated into different forms. Second, this study analyzed only communication difficulties of older adults but not those of their families or caregivers. Although we tried to contact family members and caregivers, unfortunately, few family caregivers completed the questionnaires. Thus, we could not use their counterpart data to compare communication difficulties. Third, the diagnosis of dementia was self‐reported. It is possible that cognitive impairment was underdiagnosed. Thus, we included cognitive function as an indicator of the degree of cognitive impairment. Fourth, the study was cross‐sectional. Causal relationships of communication difficulties with other variables could not be confirmed. Fifth, the sample was based on purposive sampling, and the languages of the participants were Mandarin and/or Taiwanese. The results might not be generalizable to the other older populations. Sixth, the findings and the scale may be culture‐related. Whether the findings can be generalized to other culture needs to be further examined.

## 5 Conclusions

This study assessed the dimensions of subjective communication difficulties among older adults with various degrees of cognitive impairment, factors related to communication difficulties, and associations between communication difficulties and quality of life. Older adults with impaired cognitive function may care more about negative interactions and how family/friends interact with them than about their own difficulties with cognitive function, and social support is essential to reduce communication difficulties. In the context of caregiving practices, we recommend a person‐centered approach to better understand older adults’ backgrounds and believe that spending time with them is beneficial for reducing communication difficulties. Furthermore, it is suggested that formal and informal caregivers learn how to interact with older adults with cognitive impairment to reduce their own caregiving burden. We also suggest that a longitudinal study of the relationship between cognitive function and communication difficulties among older adults and caregivers be conducted, and communication guidelines be used to examine effects of interventions designed to reduce communication difficulties in future studies.

## Author Contributions

H.‐C.H. conceptualized the study, organized the research team, obtained the funding, designed the questionnaire, analyzed the data, and wrote the manuscript. C.‐H.B., J.‐Y.W., S.‐F.L., and Y.‐W.L. conceptualized and designed the study and provided the funding. S.‐N.C.‐L. conceptualized the study, designed the questionnaire, and assisted in the investigation. All authors reviewed and approved the manuscript.

The project was supported by the National Science and Technology Council, Taiwan (NSTC 112‐2410‐H‐038‐010 to Hui‐Chuan Hsu, NSTC 112‐2410‐H‐007‐048 to Yi‐Wen Liu, NSTC 113‐2314‐B‐038‐080‐MY3 to Chyi‐Huey Bai, NSTC 112‐2410‐H‐468‐01‐SSS to Jiun‐Yi Wang, and NSTC 112‐2410‐H‐038‐014 to Sheng‐Feng Lin). The funder had no role in this study.

## Ethics Statement

The study was conducted according to guidelines of the Declaration of Helsinki. The study obtained approval from the Taipei Medical University Institutional Review Board (no. N202303043) and the Chang Gung Medical Foundation Institutional Review Board (no. 202301310B0C502).

## Conflicts of Interest

The authors declare no conflicts of interest.

## Supporting information




**Supplementary Materials**: brb371372‐sup‐0001‐FigureS1‐S3.docx

## Data Availability

According to the Institutional Review Board requirement, we do not have the right to share the data.
