# Supplementary material for: Communication Difficulties Among Older Adults With Different Degrees of Cognitive Impairment
Source: Brain Behav. 2026 Apr 6;16(4):e71372. doi: 10.1002/brb3.71372 (PMC13054135; doi:10.1002/brb3.71372)
Supplement: Supplementary file 1 — Supplementary Materials: brb371372‐sup‐0001‐FigureS1‐S3.docx [file BRB3-16-e71372-s001.docx]

Supplemental Table 1. Description of communication difficulties

| Items | Mean (SD) |
| --- | --- |
| A. You cannot speak of a word/item in your mind | 1.308 (1.067) |
| B. You often use a replacement to express what you try to say | 0.639 (0.847) |
| C. You often forget what to say during talking | 1.301 (1.022) |
| D. You feel frustrated that people do not understand your words | 0.421 (0.790) |
| E. You use gestures or facial expression when you feel difficulty to say words | 0.617 (0.850) |
| F. People often say that you repeat saying same things | 0.398 (0.816) |
| G. You pretend that you remember or know something to avoid criticism of poor memory | 0.263 (0.650) |
| H. You avoid conversation or going out because of poor memory | 0.293 (0.715) |
| I. Family/friends are not willing to talk to you | 0.278 (0.762) |
| J. Family often test your memory that makes you stressful | 0.098 (0.281) |
| K. Family or friends change their schedule for you that makes you stressful | 0.068 (0.281) |
| L. Family/friends often chat with you (reverse) | 1.180 (1.093) |
| M. You feel happy when chatting with family/friends (reverse) | 0.910 (1.033) |
| N. Family/friends try to be your company even they don’t understand your words | 1.684 (1.252) |
| O. Family understand you even you don’t speak fluently (reverse) | 1.534 (1.294) |
| P. Medical professionals or carers do not understand you | 0.120 (0.557) |
| R. Medical professionals or carers have poor attitude when talking to you or ignore you | 0.128 (0.434) |
| S. Community (e.g., neighbors, staff in shops) have poor attitude when talking to you or ignore you | 0.183 (0.553) |

| Supplemental Table 2. Factor analysis of communication difficulties | | | | | | |
| --- | --- | --- | --- | --- | --- | --- |
| Items | Communication stress | Wording difficulty | Negative interaction from professionals or community | Family/friend chatting difficulty | Family/friend adaptation difficulty | |
| Family or friends change their schedule for you that makes you stressful | **.815** | .162 | .091 | -.015 | .110 | |
| Family often test your memory that makes you stressful | **.804** | -.021 | .072 | .009 | -.100 | |
| You avoid conversation or going out because of poor memory | **.776** | .181 | .225 | .137 | -.103 | |
| People often say that you repeat saying same things | **.704** | .231 | .016 | -.182 | .068 | |
| You pretend that you remember or know something to avoid criticism of poor memory | **.608** | .180 | .152 | .245 | -.196 | |
| You often use a replacement to express what you try to say | .190 | **.801** | -.022 | .015 | -.053 | |
| You often forget what to say during talking | .252 | **.778** | .053 | .159 | .063 | |
| You cannot speak of a word/item in your mind | -.004 | **.778** | .119 | .165 | .067 | |
| You use gestures or facial expression when you feel difficulty to say words | .117 | **.690** | .039 | -.274 | -.229 | |
| Medical professionals or carers have poor attitude when talking to you or ignore you | -.208 | -.034 | **.804** | .031 | .010 | |
| Medical professionals or carers do not understand you | .172 | .038 | **.727** | .161 | -.062 | |
| Community have poor attitude when talking to you or ignore you | .205 | .261 | **.644** | .072 | -.015 | |
| Family/friends are not willing to talk to you | .372 | -.103 | **.627** | .075 | .099 | |
| You feel frustrated that people do not understand your words | .348 | .400 | **.472** | .076 | -.102 | |
| You feel happy when chatting with family/friends (reverse) | .052 | .023 | .112 | **.898** | .102 |  |
| Family/friends often chat with you (reverse) | .023 | .107 | .181 | **.874** | .194 |  |
| Family understand you even you don’t speak fluently (reverse) | -.084 | -.020 | -.028 | .062 | **.931** |  |
| Family/friends try to be your company even they don’t understand your words | -.015 | -.061 | .009 | .230 | **.910** |  |
| Note: Extracted by principle component analysis, Varimax rotation, explained variance =67.7%. | | | | | | |

Supplement Table 3. Bi-variate analysis of communication difficulties with cognitive function and dimensions

| Cognitive function and dimensions | Total communication difficulties | Communication stress | Wording difficulty | Negative interaction from professionals or community | Family/friend chatting difficulty | Family/friend adaptation difficulty |
| --- | --- | --- | --- | --- | --- | --- |
| Cognitive function | -0.269^**^ | -0.137 | -0.093 | -0.249^**^ | -0.300^***^ | -0.045 |
| Visual-space execution | -0.170 | -0.040 | 0.018 | -0.190^*^ | -0.297^**^ | -0.050 |
| Attention | -0.278^**^ | -0.064 | -0.170^*^ | -0.241^**^ | -0.335*^**^ | -0.020 |
| Calculation | -0.244^**^ | -0.165 | -0.103 | -0.218^*^ | -0.181^*^ | -0.063 |
| Language | -0.195^*^ | -0.071 | -0.013 | -0.160 | -0.226^**^ | -0.135 |
| Abstract | -0.260^**^ | -0.074 | -0.162 | -0.055 | -0.243^**^ | -0.207 |
| Recall memory | -0.098 | -0.057 | -0.070 | -0.121 | -0.090 | 0.045 |
| Time-space orientation | -0.196^*^ | -0.211^*^ | -0.058 | -0.235^**^ | -0.184^*^ | 0.075 |
| Age | 0.088 | -0.038 | 0.121 | 0.092 | 0.063 | -0.001 |
| Sex (male) | -0.018 | -0.118 | -0.004 | -0.087 | 0.034 | 0.111 |
| Education | -0.144 | 0.104 | -0.112 | -0.020 | -0.124 | **-0.238**** |
| Case source (community) | **-0.200^*^** | -0.090 | -0.091 | **-0.262^**^** | **-0.312^***^** | 0.123 |
| Other diseases | 0.145 | -0.145 | 0.163 | 0.094 | **0.280^**^** | 0.023 |
| Dementia (yes) | 0.166 | **0.291**** | **0.180*** | 0.099 | -0.074 | -0.053 |
| Subjective memory complaint (yes) | **0.252^**^** | **0.218^*^** | **0.290^**^** | 0.037 | 0.063 | 0.059 |
| Hearing (unclear) | 0.068 | -0.092 | 0.076 | -0.026 | **0.173*** | 0.059 |
| Social support | **-0.400^***^** | -0.104 | -0.114 | **-0.292^**^** | **-0.438^***^** | **-0.263**** |

Note: n=133. Reference groups: quality of life (poor), sex (female), case source (institution), dementia diagnosis (no), subjective memory complaint (no), hearing (clear); other variables were ordinal or continuous. The analysis was conducted by Pearson’s correlation. *p<0.05, **p<0.01, ***p<0.001.
